# Effects of personality traits on mindful self-care practices of healthcare workers

**DOI:** 10.4102/sajpsychiatry.v29i0.2019

**Published:** 2023-03-29

**Authors:** Iram Osman, Veena S. Singaram

**Affiliations:** 1 School of Clinical Medicine, College of Health Sciences, University of KwaZulu-Natal, Durban, South Africa

**Keywords:** coping mechanisms, health professionals, mindfulness, personality, self-care, healthcare professionals, COVID-19

## Abstract

**Background:**

Coronavirus disease 2019 (COVID-19) placed healthcare professionals (HCPs) at a higher risk for stress-related conditions. Implementing a brief online mindfulness-based intervention (MBI) was hypothesised to transform the HCPs’ ability to cope with stress by enhancing their self-care.

**Aim:**

This study aimed to explore the impact of an online MBI on HCPs’ self-care practices and determine if personality traits were a moderating variable.

**Setting:**

An online MBI was implemented for HCPs working in South Africa during the COVID-19 pandemic lockdowns.

**Methods:**

A quantitative study design included a pre-assessment and post-assessment component, which allowed paired comparison and regression analysis to confer correlations. Data were collected via two validated instruments: the Mindful Self-Care scale-2018 and the Big Five Personality test.

**Results:**

Forty-nine HCPs participated in the study. Significant improvements were found in all the major self-care subscales post-intervention (*p* < 0.05). No significant associations were found between the personality traits and self-care except for neuroticism, which appeared to be an essential moderating variable.

**Conclusion:**

An online MBI significantly impacted health professionals’ ability to care for themselves, despite their personality styles.

**Contribution:**

The impact of an online MBI on HCPs’ self-care during the most intense time of stress and with a cohort of people known to be the most vulnerable to stress, namely those with neuroticism to date, has not been commented on.

## Introduction

Coronavirus disease 2019 (COVID-19) unveiled and exponentially escalated mental health issues for many healthcare professionals (HCPs), who were already working under highly stressful conditions.^1,2,3^ The psychological impact of working with high mortality rates and reduced access to social support led HCPs to present with high levels of anxiety, depression, and post-traumatic stress.^4^ Research has demonstrated that HCPs historically exhibit poor coping mechanisms, neglect self-care, and have low help-seeking behaviours.^5,6^ Healthcare professionals working in South Africa have added challenges of working in a resource-constrained public health sector with a high rate of infectious diseases, poor infrastructure, and substantial health treatment gaps.^7^ Furthermore, South Africa implemented widescale lockdowns to curtail infection, restricting social movement. The lockdown meant that access to healthy coping mechanisms, like exercise at the gym, social gatherings, and prayer meetings, was curtailed.^8^ An increase in unhealthy coping mechanisms like substance addiction and excessive internet use was observed.^9,10^

A recent study^4^ recommended that HCPs should focus on their psychological immunity to assist them in managing unprecedented stressors. Psychological immunity is defined as adaptive coping mechanisms and personality characteristics that act as psychological buffers during stressful situations.^11^ These characteristics include reframing to see things more positively, feeling a sense of coherence, emotional regulation, and being solution-focused.^11^ Individuals who are more immune to stress can also be described as resilient. Mindfulness is a state of mind that involves paying attention with full awareness in the present moment, non-judgementally, in the service of self-understanding and wisdom.^12^ Mindfulness-based interventions (MBIs) are psychological training programmes that focus on changing individuals’ relationships to their thoughts by creating present moment focus and a non-judgemental stance,^13^ which enhances resilience.^14^ Most studies during the pandemic have focused on the adverse outcomes on mental health rather than protective variables that can promote well-being, such as mindfulness, resilience and self-care.^14^ The focus needs to be redirected to positive coping practices as these were found to be associated with a significantly lower risk of distress and psychopathology.^15^

Self-care can be defined as caring for one’s physiological and emotional needs, leading to enhanced physical, emotional, and cognitive well-being.^16^ The efficacy of self-care has been established across a range of issues among HCPs, including stress reduction, compassion for self and other individuals, burnout, psychological distress, and general health.^17,18,19^ Thus, equipping HCPs with easily accessible and timely interventions to enhance self-care would be beneficial, especially during a global pandemic. A pilot study with HCPs working in three public hospitals in South Africa during COVID-19 commented on the efficacy of an MBI on self-care.^20^ Foale et al.^20^ encouraged future MBIs to be facilitated *via* the Zoom platform to create a safe space for sharing and the need for more research because MBIs already showed such potential in the field.

However, mindfulness should not be seen as a panacea for everything, for certain factors such as personality traits could affect who is likely to benefit from these programmes. Personality traits have been found to influence how people deal with stress, coping mechanisms, and the ability to be mindful.^21^ Previous research presents the possibility that personality traits may moderate the efficacy of MBIs but warrants further investigation.^22^ It has been suggested that measuring individual characteristics may be necessary to uncover the effects of brief MBIs.^23^ However, there is a gap in the literature regarding the influence of these individual characteristics on mindfulness-based therapeutic techniques.^22^

This study explores the impact of an online MBI on HCP self-care practices and determines if personality was a moderating variable in being mindful and practising self-care. It was hypothesised that self-care practices would improve after an MBI.

## Research methods and design

### Research design

This quantitative study included a pre-assessment and post-assessment component, which allowed paired comparison and regression analysis to check for correlations.

### Setting

The study was conducted using an online platform during the first wave of the COVID-19 pandemic from June to August 2020. Lockdown level 3 was implemented at the time, restricting movement to isolate those exposed to the virus. A 1-h MBI was offered once a week for 4 weeks *via* the Zoom platform.

### Sampling

Medical interns, medical students, registrars, and other HCPs were invited *via* social media platforms, including WhatsApp groups and Facebook pages. Interested health professionals were encouraged to share the link with colleagues. Thus, purposive snowball sampling was also used. Snowball sampling has been found to work well when looking for information-rich data sources from a sample with specific characteristics.^24^ The inclusion criteria for the study included being a practising health professional working within South Africa. Informed consent was obtained from all participants before participation in the study.

### Intervention

A 4-week MBI devised by a registered clinical psychologist and mindfulness teacher was adapted for online use and facilitated by two qualified and experienced mindfulness teachers using Zoom. The MBI ran from 14 July 2020 to 05 August 2020. The intervention consisted of four 1-h group sessions; the structure is outlined in Box 1. The intervention was offered on Tuesday and Wednesday evenings at 18.00, thus if the HCPs could not join on Tuesdays, they could join the next day. The Zoom links were sent on a group chat and individually *via* emails for easy access and preference. Audio and video clips of guided meditations were provided using the WhatsApp group to supplement the interventions and encourage home practice.

BOX 1 Structure of the online mindfulness-based intervention.

**Week 1**

Welcome and introduction

Raisin exercise and inquiry

Body scan and inquiry

Input on autopilot and mindfulness (explained as text in manuals provided and using stories in session)

Poem

Three-minute breathing space and inquiry

Home practice explained (provided as videos and audios *via* WhatsApp) three minute breathing space and body scan meditation through guided meditation audios

Reflection and support (voluntary group discussions)

**Week 2**

Check-in on home practice (poll and reported challenges in chat box in Zoom)

Three minute breathing space

Review of home practice

Awareness of breath and inquiry

Yoga and inquiry

Input on breath and body awareness (explained as text in manuals provided and using stories in session)

Poem

Walking meditation and inquiry

Home practice (provided as videos and audios via WhatsApp) mindful movement yoga audio and 8-minute sitting meditation audio

Reflection and support (voluntary group discussions)

**Week 3**

Check-in on home practice (poll and reported challenges in Zoom chat box)

Three minute breathing space

Review of home practice

Awareness of thoughts and inquiry

Input on stress, acceptance, allowing, and letting be (explained as text in manuals provided and using stories in session)

Poem

Three minute breathing space and action and inquiry

Home practice (provided as audio via WhatsApp–10 min Sounds and thoughts mediation)

Reflection and support (voluntary group discussions)

**Week 4**

Check-in on home practice (poll and reported challenges *via* Zoom chat box)

Three minute breathing space

Review of home practice

Body scan and inquiry

Input on empathy, compassion, and how can I best take care of myself (explained as text in manuals provided and using stories in session)

Poem

Loving kindness meditation

### Instruments and data collection

The validated Big Five Personality Test (B5T)^25^ and Mindful Self-Care Scale-short (MSCS-2018)^26^ were used to assess personality and self-care. The MSCS-2018 was self-administered before the intervention and once again after the MBI. The B5T was provided only once before the session, as it is reported that personality is a relatively stable trait that only changes gradually over long periods.^27^

The B5T consists of 50 items on the five-point Likert-type scale, with each factor measured by 10 items and a factor analysis of over 0.7.^28,29^ This assessment was chosen because of its user-friendly scoring methods and comprehensive breakdown of the relevant personality traits. This test also showed criterion-related validity through a systematic review in South Africa.^26^ The B5T defines the personality on a continuum of extroversion, agreeableness, conscientiousness, openness to experience, and neuroticism. Extroversion reflects on the trait that seeks pleasure socially. High scorers prefer working with others, while low scorers prefer working alone. Agreeableness has to do with adjusting one’s conduct to please others. High scorers are typically obliging, while low scorers tend to speak frankly despite the opinion of others. Conscientiousness is the trait of being honest and hardworking. High scorers tend to follow the rules and prefer organisation, while low scorers may be messy and dishonest. Neuroticism is the personality trait of being prone to more negative emotions. Openness to experience is the personality trait of seeking novelty. High scorers may be more adventurous and open to change, while low scorers may be cautious.

The MSCS-2018^16^ aimed to identify self-care areas that would improve with the MBI. The MSCS-2018 is a 24-item self-reported scale that measures the frequency of self-care acts. It has six sub-domains: mindful relaxation, physical care, self-compassion and purpose, supportive relationships, supportive structures, and mindful awareness. Each item was rated on a Likert scale from 1 (never) to 5 (regularly). High scorers indicated higher levels of self-care. The mean of each sub-domain was calculated using the formula: total/number of items in the subdomain. The internal consistency coefficient of the Cronbach’s alpha (CA) for the overall scale was 0.84, comparable to other studies with a CA of 0.89.^26^

### Data analysis

Data were entered from Excel into Stata version 15.1. The data were statistically evaluated for normality of distribution, and the values presented as means with the standard deviation, unless otherwise stated. A *p-*value < 0.05 was considered statistically significant with a 95% confidence interval. The paired samples *t*-test was used to assess the mindfulness intervention’s impact on self-care. Pearson correlations and regression analysis were used to test the relationship between personality traits and self-care sub-scales.

### Ethical considerations

Individual informed consent was obtained from all participants before the study. Ethical approval (HSSREC/00000848/2019) was granted by the Humanities and Social Science Research Ethics Committee of the University of KwaZulu-Natal (UKZN).

## Results

Although 55 HCPs attended all four sessions, only 49 participants were included in the study as they completed all required pre- and post-assessments. The sample (*n* = 49) consisted of medical doctors (41%), psychologists (24%), physiotherapists (16%), occupational therapists (12%) and other allied healthcare workers (7%). Most of the participants were from public state hospitals (64%), were female (89%), and between the ages of 24 and 63 years. Experience as a HCP ranged from just a few months to 33 years.

Table 1 illustrates the self-care scores before and after intervention. The table also includes the *p*-value of the self-care scales’ differences (post and pre). The mindful awareness score measured awareness of thoughts, emotions, and bodily sensations. The mean significantly increased from 2.9 to 3.49 (*p* < 0.001). The mindful relaxation scale that measured the ability to be calm significantly improved from 2.81 to 3.24 (*p* = 0.0001). The physical self-care scales that check for necessary activities to keep the body healthy such as nutrition, hydration, and exercise significantly improved from 2.55 to 2.9 (*p* = 0.001). Self-compassion and a sense of purpose measured the ability to be reassuring to the self, view failure as part of the process, and find meaning in one’s life and work also significantly improved from a mean of 3.08 to 3.5 (*p* = 0.003). Surprisingly, supportive structures where participants felt heard and understood improved by managing their external environment. This was seen with a significant improvement of the mean from 3.48 to 3.76 (*p* = 0.02). Accessing supportive relationships enhanced from 4 to 4.2 (*p* = 0.049).

**TABLE 1:** Pre- and post-data of Mindful Self-Care Scale-short 2018.

Self-care subscales	Pre		Post		Post-pre effect size		*p*
	Mean	Standard deviation	Mean	Standard deviation	Mean	Standard deviation	
Mindful awareness	2.9	0.81	3.49	0.76	0.59	0.87	*< 0.001
Supportive structures	3.48	0.85	3.76	0.78	0.28	0.79	*0.02
Self- compassion	3.08	0.8	3.5	0.95	0.42	0.92	*0.003
Mindful relaxation	2.81	0.75	3.24	0.75	0.43	0.73	*0.0001
Supportive relationships (median, IQR)	4	(3.4 to 4.6)	4.2	(3.4 to 4.5)	0.2	(-0.2 to 0.6)	*0.049
Physical care	2.55	0.85	2.9	0.74	0.35	0.58	*0.001

IQR, interquartile range.

* *p* < 0.05 significance.

Table 2 displays the *p*-values of the post and pre-self-care scale differences as adjusted by the personality traits. Neuroticism traits appeared to be the most influential, for they significantly affected mindful awareness (*p* = 0.004), self-compassion (*p* = 0.004), and mindful relaxation (*p* = 0.02). No significant changes were found between any of the other personality traits such as extroversion, conscientiousness, agreeableness and openness to experience and the self-care subscales.

**TABLE 2:** Self-care subscales difference (post-pre) adjusting for personality traits.

Self-care subscales difference (post/pre)	Agreeableness	Extraversion	Conscientiousness	Neuroticism	Openness to experience
Mindful awareness	0.64	0.05	0.27	*0.004	0.5
Supportive structures	0.61	0.47	0.56	0.054	0.34
Self- compassion	0.1	0.29	0.19	*0.004	0.6
Mindful relaxation	0.64	0.74	0.69	*0.02	0.3
Supportive relationships	0.23	0.47	0.76	0.06	0.6
Physical care	0.94	0.25	0.37	0.43	0.51

* *p* < 0.05 significance.

## Discussion

This study aimed to explore the impact of a brief online MBI on HCP self-care practices and determine if the big five personality traits (extroversion, agreeableness, conscientiousness, openness to experience, and neuroticism) were moderating variables in being mindful and practising self-care. Healthcare professionals were thrust into the frontline of a global health crisis that overwhelmed them and rendered many feeling helpless.^8^ Psychological immunity was required at this time to show fortitude in the face of uncertainty and high distress levels.^4^ Building psychological immunity involves various traits such as mindful acceptance, good social support, a sense of coherence, emotional regulation, and positive self-efficacy,^11^ which the MBI attempted to augment. This will now be explored in greater depth based on the MSCS-2018 instrument used in this study.

The most critical point for this study to be deemed effective was to evaluate whether a meaningful increase in mindful awareness was observed after attending the MBI. The mindful awareness scale significantly increased post-intervention, confirming the efficacy of the MBI. Each component of the definition of mindfulness as described by Kabat-Zinn^12^ is hypothesised to facilitate the improvement observed in the HCP’s self-care, for example, the non-judgemental aspect. The concept of non-judgement entails not classifying things as good or bad but accepting them just as they are in the present moment without reacting based on past experiences. Instilling the non-judgemental accepting stance of present-moment experience has been found by prior research to result in transformative health behaviour such as healthy eating, exercise, and a reduction in substance use because of being able to better manage their stress.^12^ Similarly, improvements were observed in this study in the subscale of physical care, mindful relaxation, and supportive structures. Supportive structures consisted of questions examining the HCPs’ ability to manage a work-life balance. These subscales were critical because if HCPs are prone to overworking and do not prioritise time for themselves, they are at higher risk of psychological distress and burnout.^30^ However, balancing work and recreation leads to a healthier workforce and, subsequently, better patient outcomes.^31^

Kabat-Zinn^12^ also referred to acceptance in the service of self-understanding in his definition of mindfulness. Being self-compassionate has been described as being kind to oneself when faced with personal inadequacies or difficult situations, understanding life’s struggles in common humanity, and allowing a full range of emotions to be experienced without judgement.^32^ Many studies have shown mindfulness to enhance self-compassion, which makes for a healthier relationship to self and better personal and professional relationships.^32^ In turn, supportive social ties further mitigate trauma symptoms and enhance stress resiliency.^32^

In terms of personality, this study found that people with neuroticism appeared to benefit more from the MBI. However, the relationship between mindfulness and neuroticism is complex. Some studies show that higher levels of neuroticism negatively impact one’s willingness to participate in MBIs and ability to be mindful.^30,31,32^ While others report that high neuroticism can benefit more from MBIs^19,33^ over time.^34^ A reason hypothesised for the MBI working well with neuroticism is that an efficient stress reduction intervention was offered to a group of people prone to stress^35^ who often did not realise the range of resources available to them in terms of stress reduction,^36^ when it was needed the most. Even though the relationship between neuroticism, mindfulness and stress is complex, the findings of this study highlighted the potential merits of the MBI to help the HCPs take better care of themselves, despite their personality traits.

To summarise, it was found that the brief online MBI that was implemented in this study positively impacted HCPs’ mindful awareness and self-care levels, creating a sense of psychological immunity during the global pandemic. This impact was so significant that even the neuroticism personality trait, known for negatively impacting the ability to be mindful, did not deter the psychological immunity in this study. The neurotic participants appeared to benefit more from the MBI, for they were better able to relate with compassion and calm. This is imperative for HCPs because it is difficult to care for others if one cannot take care of oneself. This realisation is critical for HCPs not just to survive but thrive in times of uncertainty and distress.

### Limitations

This timing and subsequent setting of the study came with some unavoidable limitations. For example, the gold standard of quantitative studies would be double-blind, randomised placebo-controlled designs.^37^ The advantage of this design, would be to rule out any sample bias. However, because of the nature and timing of this study during a global pandemic, this was not feasible.

There is no single definition of mindfulness on which all researchers have agreed on, thus the measurement of mindfulness poses conceptual and methodological challenges.^37^ Assessments that are used for mindfulness often require self-reported measures. However, the more mindful participants become, the more their awareness grows on how busy the mind can be especially in the short term.^37^ This could negatively impact the assessment scores.

The MBI was offered over a month with assessments being carried out pre- and post-intervention. Thus, a limitation is that there was no follow up after that. This brings up a question of whether the intervention might have only transient and short-term effects. A follow-up after a month was set up to give feedback to participants and provide a check-in for mindfulness practice. Although, there were no quantitative assessments repeated, qualitatively and by way of polls it was observed that HCPs were still reaping the benefits of the MBI.

### Future directedness

Dropout rates are challenging in intervention studies and fall between 15% and 20%.^38^ The dropout rate for this study was negligible at 11%, and an interesting observation in this study was the higher adherence and lower dropout rate than with in-person interventions.^39^ To further reduce the dropout rate, increase accessibility, and enhance convenience would be for the MBI to be offered within working hours and supported by the relevant departments.

The results of this study indicated that increased self-care and enhanced healthier coping mechanisms, could greatly benefit medical professionals. The online MBI offered in this study may have the potential for employees as part of a wellness programme, as well as for health professional students, by improving coping mechanisms and awareness of self-care.

## Conclusion

A brief online MBI was found to significantly impact health professionals’ ability to care for themselves and healthily cope with stressors, despite their personality styles. This study found that neurotic participants appeared to benefit more from the MBI, which showed its efficacy in a highly stressful time and space.
